# Skin cytokine expression in patients with fibromyalgia syndrome is not different from controls

**DOI:** 10.1186/s12883-014-0185-0

**Published:** 2014-09-22

**Authors:** Nurcan Üçeyler, Susanne Kewenig, Waldemar Kafke, Sarah Kittel-Schneider, Claudia Sommer

**Affiliations:** Department of Neurology, University of Würzburg, Josef-Schneider-Str. 11, 97080 Würzburg, Germany; Department of Psychiatry, University of Würzburg, Füchsleinstr. 15, 97080 Würzburg, Germany

**Keywords:** Fibromyalgia syndrome, Skin biopsy, Monopolar depression, Cytokines, Opioid receptor

## Abstract

**Background:**

Fibromyalgia syndrome (FMS) is a chronic pain syndrome of unknown etiology. There is increasing evidence for small nerve fiber impairment in a subgroup of patients with FMS. We investigated whether skin cytokine and delta opioid receptor (DOR) gene expression in FMS patients differs from controls as one potential contributor to small nerve fiber sensitization.

**Methods:**

We investigated skin punch biopsies of 25 FMS patients, ten patients with monopolar depression but no pain, and 35 healthy controls. Biopsies were obtained from the lateral upper thigh and lower calf. Gene expression of the pro-inflammatory cytokines tumor necrosis factor-alpha (TNF), interleukin (IL)-6, and IL-8 and of the anti-inflammatory cytokine IL-10 was analyzed using quantitative real-time PCR and normalizing data to 18sRNA as housekeeping gene. Additionally, we assessed DOR gene expression.

**Results:**

All cytokines and DOR were detectable in skin samples of FMS patients, patients with depression, and healthy controls without intergroup difference. Also, gene expression was not different in skin of the upper and lower leg within and between the groups and in FMS patient subgroups.

**Conclusions:**

Skin cytokine and DOR gene expression does not differ between patients with FMS and controls. Our results do not support a role of the investigated cytokines in sensitization of peripheral nerve fibers as a potential mechanism of small fiber pathology in FMS.

## Background

Fibromyalgia syndrome (FMS) is a chronic pain condition that is regularly associated with additional symptoms like depressive mood, fatigue, and sleep disturbance [1]. The pathophysiology of pain in FMS is incompletely understood. In a recent study we showed that small nerve fiber impairment is present in a subgroup of FMS patients [2]; these results were confirmed by others [3-6]. The reason for small fiber pathology in FMS is unclear, and the question is if local factors may contribute to nerve fiber damage, fiber sensitization, and pain.

Cytokines are pleiotropic proteins that are key players in pain induction and maintenance [7]. In the last years cutaneous cytokine expression has been investigated in different neuropathic pain conditions. We found that in affected skin samples of patients with length-dependent idiopathic small fiber neuropathy the gene expression of some pro-inflammatory cytokines is higher compared to unaffected skin and that cytokine gene expression in the distal skin of these patients is also higher compared to healthy controls [8] while no such differences could be found in patients with Fabry disease and associated small fiber impairment [9]. Systemic cytokine expression has extensively been investigated in patients with FMS, for review see [10], however, so far no consistent pattern has arisen. In skin of FMS patients, but not of healthy controls, gene expression of the pro-inflammatory cytokines tumor necrosis factor-alpha (TNF), interleukin (IL-)1beta, and IL-6 was reported, using reverse transcription PCR [11]. The same group also reported an increased expression of skin opioid receptors in FMS patients compared to controls [12].

Here we investigated cutaneous gene expression of pro- and anti-inflammatory cytokines and of the delta opioid receptor (DOR) in skin samples of patients with FMS and compared our results with those of patients with monopolar depression but without pain as disease controls and with healthy controls. We hypothesized that patients with FMS have a higher cutaneous gene expression of pro-inflammatory, algesic cytokines as the basis of peripheral nerve fiber sensitization and that DOR expression is reduced leading to an impaired effect of endogenous opioids with increased pain.

## Methods

### Patients and controls

Between 2007 and 2011 we prospectively recruited 25 patients with FMS (23 women, two men; median age: 59 years, range: 50–70). The diagnosis was made according to the 1990 criteria of the American College of Rheumatology (ACR) [13]. Patients were recruited from all over Germany mainly by contacting self-help organisations. After a telephone interview suitable patients were invited to the Department of Neurology and were examined neurologically and electrophysiologically to check the in- and exclusion criteria as described earlier [2]. Twenty-five patients could be included. Our inclusion criteria were: male and female patients >18 years of age; medically confirmed diagnosis of FMS according to the 1990 ACR criteria; other possible differential diagnoses excluded (e.g. rheumatologic, orthopaedic). Exclusion criteria were: other differential diagnoses explaining the pain (e.g. rheumatologic, orthopaedic); other and additional pain sources (e.g. pain due to arthritis); abnormalities in routine blood tests.

Depressive symptoms are frequently present in FMS patients [1]. To control for possible confounding effects of depression on study results, we additionally investigated a group of ten patients (nine women, one men; median age: 50 years, range 39–75) with monopolar major depression without pain. These patients were enrolled between 2010 and 2011 at the Department of Psychiatry at the University of Würzburg. An additional group of 35 healthy controls was recruited. The group consisted of 15 men and 20 women with a median age of 51 years (20–84 years). Our study was approved by the Würzburg Medical School Ethics Committee. Written informed consent was obtained from all study participants before enrolment. Data on clinical examination, examination with electrophysiological measurements, pain and depression questionnaires have been published elsewhere [2].

### Skin punch biopsies

Five-mm diameter skin punch biopsies (Stiefel, Offenbach, Germany) were obtained for histological analysis and for quantitative real-time PCR (qRT-PCR) analysis as previously described [8]. Two biopsies were taken from each participant (lateral proximal thigh and distal calf). One patient with FMS refused a biopsy at the thigh and another patient refused skin punch biopsy at all. Skin samples were divided in two pieces. One was processed for immunohistochemical analysis to determine intraepidermal nerve fiber density. Methodology and results are presented elsewhere [2]. The second half was flash-frozen in liquid nitrogen and stored at −80°C before further processing for gene expression analyses.

### RNA extraction from skin samples

RNA extraction followed a protocol described earlier [8]. After thawing skin samples were immersed in 1 ml TRIzol reagent (Invitrogen, Karlsruhe, Germany), and dispersed (Polytron 1600E, Luzern, Switzerland). Samples were then incubated in 200 μl chloroform (25°C, 3 minutes) and were centrifuged (12.000 g, 15 minutes, 4°C). Supernatants were mixed with 500 μl of isopropanol and incubated again (25°C, 10 minutes). After another centrifugation step (12.000 g, 10 minutes, 4°C), the pellet was washed with 75 % ethanol and spun again (7,500 g, 5 minutes, 4°C). The samples were air-dried and the pellet was dissolved in diethylpyrocarbonate-treated water. Afterwards, samples were incubated in a water bath (55°C, 10 minutes).

### Reverse transcription PCR

All PCR reagents and cyclers were purchased from Applied Biosystems (Darmstadt, Germany). Extracted mRNA (500 ng) was reverse transcribed using TaqMan Reverse Transcription Reagents. Using 10 μl 10× PCR-buffer, 6.25 μl Multiscribe reverse transcriptase, 2 μl RNase inhibitor, 22 μl MgCl_2_, 20 μl dNTPs the reactions were performed in the ABI PRISM 7700 Cycler at the following conditions: 25°C, 10 min; 48°C, 60 min; 95°C, 5 min.

### Gene expression analysis

TaqMan Universal Master Mix and 5 μl of cDNA were used for qRT-PCR performed in the GeneAmp 7700 sequence detection system with the following gene specific TaqMan Assays: TNF (ID: Hs00174128_m1), IL-6 (ID: Hs00174131_m1), IL-8 (ID: Hs00174103_m1), and IL-10 (ID: Hs00174086_m1). Additionally, we assessed gene expression of DOR (ID: Hs00538331_m1). 18sRNA (ID: Hs99999901_s1) served as endogenous control. The 25 μl-reaction mix contained 12.5 μl TaqMan Master Mix and 1.25 μl primer. The cycler conditions were: 50°C, 2 min; 95°C, 10 min; 45 cycles with 95°C, 15 sec; 60°C, 1 min. Each plate contained a negative control and for blood measurements in addition a calibrator sample, which was the blood sample of the control person whose threshold cycles (Ct)-values were next to the calculated mean of all control blood samples measured for each primer. Samples were measured as triplicates, except for 18sRNA-values tested as duplicates. All plates were analyzed applying identical conditions. We used the comparative ∆Ct-method (i.e. relating target gene expression with individual 18sRNA expression) for individual assessment. Lower ∆CT values (i.e. sample detection at earlier PCR cycles) indicate higher gene expression. In addition, we compared gene expression in the patient and control groups using the ∆∆Ct-method as previously described [14]. Here, the above mentioned ∆Ct-value is related to a calibrator sample which is the skin sample from the healthy control group with a Ct-value next to the group mean.

### Statistical analysis

IBM PASW Statistics 21 software (IBM, Ehningen, Germany) was used for statistical analysis. For data comparison of non-normally distributed data the non-parametric Mann–Whitney-U-test was applied. Data distribution was tested with the Kolmogorov-Smirnov-test and by observing data histograms. Results of non-normally distributed data are given as median and range. P < 0.05 was considered significant.

## Results

### Cytokine gene expression does not differ between FMS patients and controls

**Figure 1:**
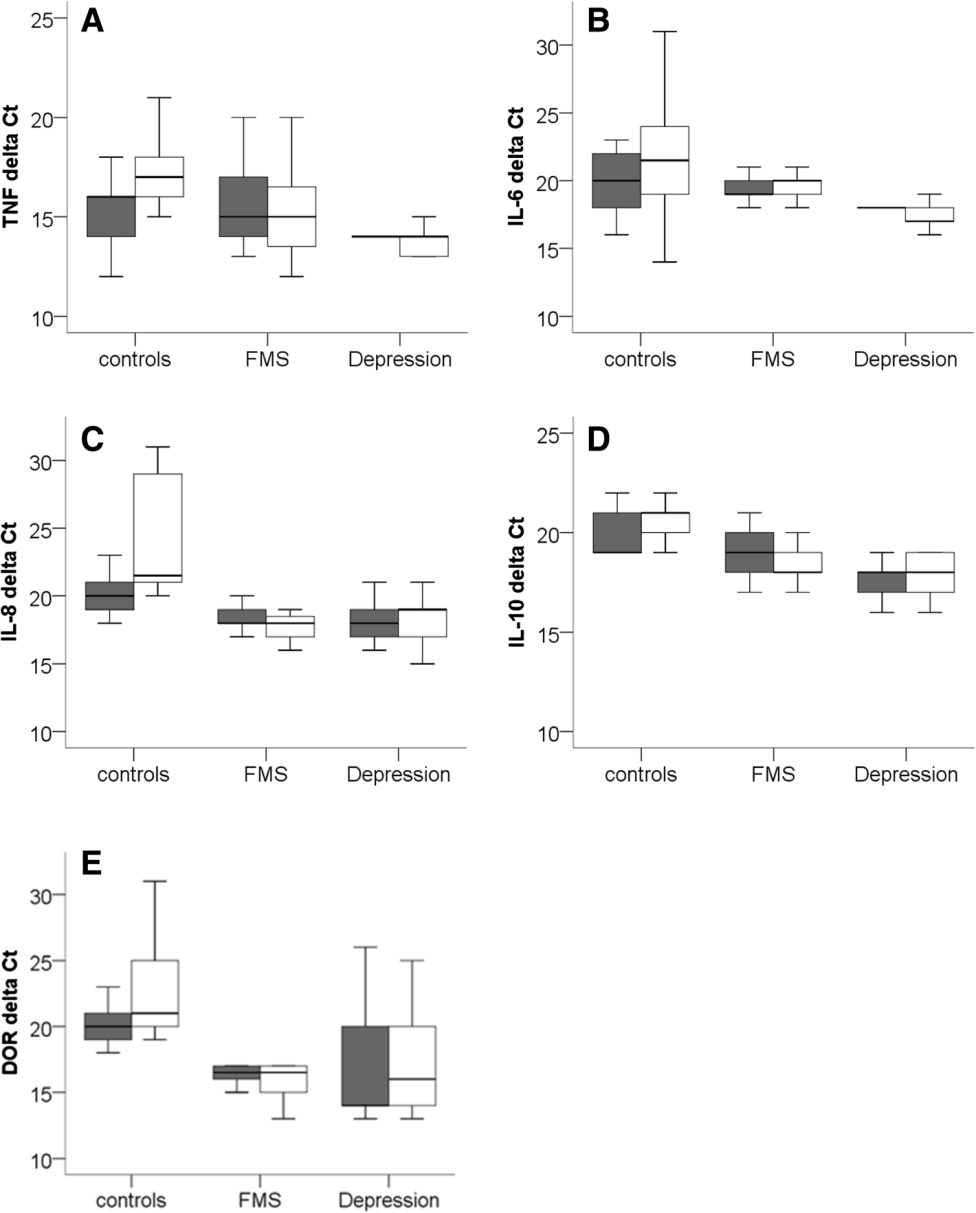
**Boxplots show ΔCT values, i.e. relation of the CT value of the target normalized to the housekeeping gene 18sRNA, of tumor necrosis factor-alpha (TNF, A), interleukin (IL)-6 (B), IL-8 (C), and IL-10 (D) and of the delta opioid receptor (DOR) (E) in skin samples of the upper thigh (grey boxes) and the lower leg (white boxes) of patients with fibromyalgia syndrome (FMS), monopolar depression without pain, and healthy controls.** Lower ΔCt values indicate higher gene expression (i.e. earlier target copy detection). No intergroup difference was found for any of the investigated targets.

Table 1 gives baseline data of the patient groups. The investigated pro- and anti-inflammatory cytokines TNF, IL-6, IL-8, and IL-10 as well as DOR were expressed in skin from the upper and lower leg of patients with FMS, depression, and healthy controls (Figure 1). No difference was found when comparing gene expression between groups (Figure 1) and of the upper and lower leg (data not shown).

**Table 1:** **Basic data of patient groups**

	**FMS**	**Depression**	**Healthy controls**
M, F	2, 23	1, 9	15, 20
Median age (range) [yrs]	59 (50–70)	50 (39–75)	51 (20–84)
Median disease duration (range) [yrs]	21 (3–50)	23 (3–35)	Not applicable

### FMS subgroups do not differ with regard to skin cytokine gene expression

As published earlier [2] the intraepidermal nerve fiber density was reduced in a subgroup of the FMS patients. When we divided the FMS group in patients with normal skin innervation and patients with abnormal distal or distal and proximal intraepidermal nerve fiber density, we did not observe a difference in cytokine and DOR gene expression between subgroups. Also, no correlation was found between current pain intensity and skin cytokine or DOR gene expression (data not shown).

## Discussion

In this study we investigated the local gene expression of selected pro- and anti-inflammatory cytokines and of DOR in skin biopsies of patients with FMS, with monopolar depression, and of healthy controls. In contrast to our hypotheses no increase in skin cytokine and no decrease in skin DOR gene expression was found in FMS patients compared to patients with depression and to healthy controls.

Systemic cytokine gene and protein expression has been investigated extensively in FMS patients giving contradictory results [10]. The hypothesis of the majority of these studies is an increase in pro-inflammatory and algesic cytokines in FMS patients as one contributor to pain. The so far only study investigating skin cytokine expression in FMS patients was published by Salemi et al. [11]. The group used reverse transcription PCR and reported the presence of the pro-inflammatory cytokines IL-1beta, IL-6, and TNF in skin from the deltoid region of FMS patients only while this was absent in healthy controls [11]. This finding was interpreted in terms of expression of algesic cytokines in skin of FMS patients contributing to pain generation and maintenance. In the current study we used qRT-PCR and show that pro- and anti-inflammatory cytokines are not only present in skin of patients with FMS but also in patients with depression and healthy controls. The finding of cytokine expression in healthy skin is in accordance with previous reports [8,9]. One reason for the discrepancy in results apart from the difference in biopsy sites compared to the data of Salemi et al. may be that qRT-PCR is the more sensitive method. In a study investigating TNF immunoreactivity on formaline fixed skin biopsy samples of patients with FMS and healthy controls also no intergroup difference could be found [15].

In a second study Salemi et al. investigated DOR and mu-opioid receptor gene expression in skin samples of FMS patients compared to healthy controls. In this study qRT-PCR was used [12]. Here, the authors reported an almost 60fold higher gene expression in skin samples of FMS patients compared to controls. Information on the region of biopsy and the used primers were not provided so that a direct comparison with our data obtained from the upper and lower leg is not possible. Also, the methods of data comparison between Ct values of FMS patients and the control groups was different in both studies, which may be the major reason for this discrepancy, since already small differences in Ct-values may appear large when transferred to x-fold changes.

In our previous study we showed that small fiber impairment is present in a subgroup of patients with FMS [2]; our findings were confirmed by others [3-6]. The open question is how reduced intraepidermal nerve fibers may be linked with peripheral hyperalgesia and pain. One possibility is that the remaining peripheral nerve endings are sensitized by local influences such as pro-inflammatory and algesic cytokines. However, we did not find a positive support for this assumption in our study and suggest that other local factors like chemokines need to be investigated and also the ion channel repertoire of the remaining supposedly diseased nerve fibers.

Our study has several limitations. The number of patients was small and with the relatively high variation of the individual qRT-PCR values potential group differences may have been masked. The lack of difference in gene expression patterns does not necessarily mean lack of difference in the actually functional protein levels, however, we were not able to conduct additional protein analyses due to the limited amount of bio material. This was also the reason for the selected panel of investigated targets. Also, the assessment of whole skin samples instead of separated skin cells may be another reason why potentially present differences between groups may have been missed.

## Conclusion

We conclude that cytokine gene expression is not restricted to FMS skin and that for the investigated cytokines there is no intergroup difference that might have been a plausible factor contributing to intraepidermal nerve fiber sensitization. The possibility of a ganglionic impact on fiber hyperexcitability and of other potential mechanisms of peripheral nerve sensitization need to be considered as well as other algesic mediators in FMS skin.

## Abbreviations

ACR: American college of rheumatology
DOR: Delta opioid receptor
FMS: Fibromyalgia syndrome
IL: Interleukin
PCR: Polymerase chain reaction
TNF: Tumor necrosis factor-alpha

## References

[1] Häuser W, Zimmer C, Felde E, Kollner V (2008). What are the key symptoms of fibromyalgia? Results of a survey of the German Fibromyalgia Association. Schmerz (Berlin, Germany).

[2] Üçeyler N, Zeller D, Kahn AK, Kewenig S, Kittel-Schneider S, Schmid A, Casanova-Molla J, Reiners K, Sommer C (2013). Small fibre pathology in patients with fibromyalgia syndrome. Brain.

[3] Oaklander AL, Herzog ZD, Downs H, Klein MM (2013). Objective evidence that small-fiber polyneuropathy underlies some illnesses currently labeled as fibromyalgia. Pain.

[4] Serra J, Collado A, Sola R, Antonelli F, Torres X, Salgueiro M, Quiles C, Bostock H (2014). Hyperexcitable C nociceptors in fibromyalgia. Ann Neurol.

[5] Giannoccaro MP, Donadio V, Incensi A, Avoni P, Liguori R (2013). Small nerve fiber involvement in patients referred for fibromyalgia. Muscle Nerve.

[6] de Tommaso M, Nolano M, Iannone F, Vecchio E, Ricci K, Lorenzo M, Delussi M, Girolamo F, Lavolpe V, Provitera V, Stancanelli A, Lapadula G, Livrea P (2014). Update on laser-evoked potential findings in fibromyalgia patients in light of clinical and skin biopsy features. J Neurol.

[7] Austin PJ, Moalem-Taylor G (2010). The neuro-immune balance in neuropathic pain: involvement of inflammatory immune cells, immune-like glial cells and cytokines. J Neuroimmunol.

[8] Üçeyler N, Kafke W, Riediger N, He L, Necula G, Toyka KV, Sommer C (2010). Elevated proinflammatory cytokine expression in affected skin in small fiber neuropathy. Neurology.

[9] Üçeyler N, He L, Schönfeld D, Kahn AK, Reiners K, Hilz MJ, Breunig F, Sommer C (2011). Small fibers in Fabry disease: baseline and follow-up data under enzyme replacement therapy. J Peripher Nerv Syst.

[10] Üçeyler N, Häuser W, Sommer C: **Systematic review with meta-analysis: cytokines in fibromyalgia syndrome.** *BMC Musculoskelet Disord* 2011, **12:**245. 10.1186/1471-2474-12-245 PMC3234198 22034969

[11] Salemi S, Rethage J, Wollina U, Michel BA, Gay RE, Gay S, Sprott H (2003). Detection of interleukin 1beta (IL-1beta), IL-6, and tumor necrosis factor-alpha in skin of patients with fibromyalgia. J Rheumatol.

[12] Salemi S, Aeschlimann A, Wollina U, Gay RE, Michel BA, Gay S, Sprott H (2007). Up-regulation of delta-opioid receptors and kappa-opioid receptors in the skin of fibromyalgia patients. Arthritis Rheum.

[13] Wolfe F, Smythe HA, Yunus MB, Bennett RM, Bombardier C, Goldenberg DL, Tugwell P, Campbell SM, Abeles M, Clark P, Fam AG, Farber SJ, Fiechtner JJ, Franklin CM, Gatter RA, Hamaty D, Lessard J, Lichtbroun AS, Masi AT, Mccain GA, Reynolds WJ, Romano TJ, Russell IJ, Sheon RP (1990). The American college of rheumatology 1990 criteria for the classification of fibromyalgia. Report of the multicenter criteria committee. Arthritis Rheum.

[14] Winer J, Jung CK, Shackel I, Williams PM (1999). Development and validation of real-time quantitative reverse transcriptase-polymerase chain reaction for monitoring gene expression in cardiac myocytes in vitro. Anal Biochem.

[15] Blanco I, Beritze N, Arguelles M, Carcaba V, Fernandez F, Janciauskiene S, Oikonomopoulou K, de Serres FJ, Fernandez-Bustillo E, Hollenberg MD (2010). Abnormal overexpression of mastocytes in skin biopsies of fibromyalgia patients. Clin Rheumatol.

